# Conflict among Iranian hospital nurses: a qualitative study

**DOI:** 10.1186/1478-4491-7-25

**Published:** 2009-03-20

**Authors:** Nahid Dehghan Nayeri, Reza Negarandeh

**Affiliations:** 1 School of Nursing and Midwifery, Tehran University of Medical Sciences, Tehran, Iran

## Abstract

**Background:**

This study aims to explore the experience of conflict as perceived by Iranian hospital nurses in Tehran, Islamic Republic of Iran. Although conflict-control approaches have been extensively researched throughout the world, no research-based data are available on the perception of conflict and effective resolutions among hospital nurses in Iran.

**Methods:**

A qualitative research approach was used to explore how Iranian hospital nurses perceive and resolve conflicts at work. A purposive sample of 30 hospital nurses and nurse managers was selected to obtain data by means of in-depth semi structured interviews. Data were analysed by means of the content analysis method.

**Results:**

The emerging themes were: (1) the nurses' perceptions and reactions to conflict; (2) organizational structure; (3) hospital management style; (4) the nature and conditions of job assignment; (5) individual characteristics; (6) mutual understanding and interaction; and (7) the consequences of conflict. The first six themes describe the sources of the conflict as well as strategies to manage them.

**Conclusion:**

How nurses perceive conflict influences how they react to it. Sources of conflict are embedded in the characteristics of nurses and the nursing system, but at the same time these characteristics can be seen as strategies to resolve conflict. We found mutual understanding and interaction to be the main factor able to prevent and resolve conflict effectively. We therefore recommend that nurses and nurse managers encourage any virtues and activities that increase such understanding and interaction. Finally, as conflict can destroy individual nurses as well as the nursing system, we must act to control it effectively.

## Background

Conflict is one of many issues found in any organization, including hospitals, where constant human interaction occurs [1,2]. The potential for conflict to arise in a hospital setting is considerably higher due to the complex and frequent interactions among the nurses and other employees and the variety of roles they play. Specialization and organizational hierarchy often add to the territorial conflicts in hospitals [3,4]. Although a reasonable amount of conflict in the form of competition can contribute to a higher level of performance and a conflict-free work environment is an exception, how conflict is addressed is of paramount importance [5]. The sources of conflict among hospital nurses and health care personnel include authority positions and hierarchy, the ability to work as a team, interpersonal relationship skills, and the expectations of performing in various roles at various levels [6].

Researchers believe that functional conflict can turn into emotional conflict if not managed properly, which in turn disrupts collaborative efforts [7]; leads to unprofessional behaviors [8]; results in under commitment to the organization [9]; increases psychological stress [10] and emotional exhaustion [11,12]; results in mistreatment of patients [12]; elevates anxiety and work resignation [13]; and decreases altruistic behaviors [14]. This is only a short list of negative consequences of poorly managed conflict. Nevertheless, some researchers argue that conflict, if treated with wisdom and creativity can result in positive performance in the organization [15]. Finally, conflict influences clinical decision-making as much as collaboration and positive relationships do [7].

The first step for the effective management of conflict would be the recognition of conflict and its sources from the viewpoints of nurses/caregivers and then understanding how to moderate and control them according to those viewpoints [16,17]. Once the conflict and its source are identified, addressing the conflict would be instrumental in enhancing professional development and reducing the burnout rate among nurses [2].

A literature review points to the paucity of information relevant to this study and reveals many studies from industrial and political entities. Considering how much hospital and industrial settings differ, the suggested strategies seem inadequate for conflict resolution among hospital nurses. Our experiences as a clinical nurse, nurse manager and researcher indicate that conflict is a daily problem in the hospital setting, especially for nurses. Therefore, we conducted an inquiry to explore Iranian hospital nurses' experiences with conflict in the hospital setting. We aimed to identify the sources of conflict and how nurses and nurse managers deal with conflicts daily.

### Health care in Iran

The Islamic Republic of Iran is a country of 70 million people, more than two thirds of whom are under the age of 30. Culturally, Iranians are Muslims (98%); their official language is Farsi, or Persian. According to the World Health Organization, Iran's literacy rate is 82%; life expectancy for men is 70 years and 73 years for women [18].

In Iran until 1915, hospitalized patients received care from untrained personnel. Subsequently foreign missionaries came to Iran, and as they performed their religious duties they introduced the modern form of nursing and provided health care services. Missionaries trained a small number of Iranian women to care for hospital patients.

In 1916, the first three-year nursing programme was established, in the city of Tabriz. Currently there are approximately 70,000 nurses employed in the Iranian health care system. Male and female nursing students are enrolled at various universities to study nursing at the bachelor's to doctoral level. Today, nursing in Iran is a recognized profession with its own Nursing Organization of the Islamic Republic of Iran (NOIRI), founded in 2000. This organization is charged to improve and promote the Iranian nursing profession.

## Methods

A qualitative research method was used to explore sources of conflict for nurses and nurse managers and how they handle it in daily practice. Thirty hospital nurses and nurse managers were selected purposively and interviewed by the first researcher with aim of capturing their experiences in the area of conflict on the job. The inclusion criterion for staff members was a minimum of three years' work experience. After giving their informed consent, nurses and nurse managers were given an appointment according to their schedule and preferred date and time. The time and place were planned according to the participant's preference in a private place in the ward. Each interview began with a broad question, such as: "Could you explain your experiences with conflict?", or, "Tell me about how you have resolved a conflict in the past". The interviews lasted between 40 and 75 minutes, but on the average it took one hour if the participant was interested in elaborating on his or her experience. Interviews were tape-recorded and transcribed verbatim.

Content analysis was based on scrutiny of the transcripts. Meaningful segments of data were identified and coded with appropriate labels in the transcribed text. These codes were clustered under the categories of sources of conflict and the ways in which participants managed conflict, by means of comparative analysis. For example, participants 2, 6, 13, 24 and 26 expressed disjuncture between how they conceived their role and what they actually did, which we categorized under "the nature and conditions of the job". Similarly, numerous participants spoke about the effect of conflict on nurses' physical and spiritual health. Concurrent analysis and sampling continued until saturation was reached and researchers arrived at a meaningful description of what was occurring among nurses regarding conflict. This took place after 30 interviews.

Trustworthiness and data credibility were established via face-to-face discussions with individual participants and fellow researchers and by prolonged engagement. The researcher made every effort to clarify participants' perceptions and the emergent themes to determine whether the codes and themes identified were appropriate to their experiences. The participants were contacted for verification of analysed data from the full interview transcript and the summary. Maintaining long-term communication with the participants helped the researcher to establish trust and reach a better understanding of participants in the field.

Three faculty members served as peer reviewers to ensure that no data were lost in transcription and content analysis. If any disagreement occurred, group discussion was conducted to let them to reach general agreement. Approximately 60% of the transcripts, codes and categories were rechecked for group consensus

### Ethical considerations

The research proposal was approved by the Tehran University of Medical Sciences Research Committee. All participants were informed about the purpose of the study and assured of confidentiality and anonymity. Participants signed an informed consent indicating that their participation in this study was voluntary and without any obligation to continue.

## Results

Among the 30 staff members and nurse managers who participated in the study, there were 19 nurses, five head nurses, four supervisors and two nurse managers (matrons). All the participants worked in various wards – such as orthopaedics, neonatal intensive care, intensive care, medicine, obstetrics, urology, coronary care – and the emergency department at university hospitals in Tehran. The participants' ages ranged from 28 to 56 years, with a mean age of 36.5). The nurses' experience ranged from three to 28.5 years, with a mean of 14 years. Twenty-six participants were female and four were male. Twenty-eight had bachelor's degrees and two had master's degrees.

Seven themes were identified during the data analysis process: (1) the nurses' perception of and reaction to conflict; (2) organizational structure; (3) hospital management style; (4) the nature and conditions of job assignment; (5) individual characteristics; (6) mutual understanding and interaction; and (7) the consequences of conflict.

### The nurse's perception and reaction to conflict

Participants interpreted conflict as any form of verbal aggression, disagreement, discrimination, psychological stress, interpersonal differences, violence, anger and non-coping behaviour. Some participants perceived conflict as the disparity between expectations and realities.

Different views were expressed regarding the existence and control of conflict among nurses. Some participants believed that there not should be any conflict in nursing as a humanistic profession. Others contended that conflict cannot be eliminated and is a normal occurrence in every work environment. Several participants shared that conflict emanates mainly from an individual's behaviour and personality, while the majority of participants believed in multiple sources of conflict. For example, one of the participants said:

"It seems to me conflict means everything that we expect from nursing and then we saw what they expected from us as a nurse."

"The first thing that comes to my mind about conflict is two contrary things or people."

The types of reaction to conflict also varied according to the participant's perception of conflict. Reactions such as anger and aggression, shouting at team members and colleagues, a tearful feeling of resignation and sorrow, apology, self-control, calming behaviour, forgiveness, flexibility and coping with oneself were enumerated by the participants.

About ways of reacting, participants said:

"If I experience conflict with my colleague I would try to ignore it, if possible, whereas if it was severe enough that I felt it hurt me, I would warn them."

"the other day ... I faced a lot of stress, so I got a nervous breakdown ... I had the feeling of going home and starting to yell and shout to get everything off my mind... or to confide in my family..."

### Organizational structure

Participants pointed out some of their experiences with conflict in the workplace. One of the recurring criticisms related to the hospital affiliation with the universities (teaching hospitals) was the slow process of management, numerous and redundant medical orders written by medical interns, residents and attending physicians and the presence of unskilled and inexperienced medical students contributed to the rising level of conflict.

A subcategory of this variable is the hospital facilities. Budget deficits, the hospitals' self-governance policy and the lack of sufficient medical equipment and medicines created much stress and conflicts for the patients, families and staff.

"All the companions of the patient demand more care for their patients and when they are told about the lacks, shortages and inadequacies of facilities they turn a deaf ear to us. This has often led to severe conflicts."

In addition, inadequate facilities, improper functioning of other departments and neglected responsibilities created pressure and conflict among the personnel. These inadequacies eventually reduced the tolerance threshold, which in turn contributed to the conflict experienced.

"Too much pressure on this shift... Scanty facilities... very meager...you feel really exhausted...amounting to tensions and conflicts which are often displaced onto people around...you know...yelling at colleagues..."

The workforce structure is another subcategory regarded by participants as having a significant role in causing and controlling conflicts. An excessive number of patients, lack of personnel, failure to recruit new personnel according to standards and obligatory overtime work left nurses feeling angry, violated and exploited without any control over the situation. Participants believed that unskilled staff failed to meet patients' needs, harmed the patient-nurse relationship and damaged staff morale. Meanwhile, the patients expected good nursing care and no one could explain the situation for them.

The individual and cultural characteristics of the patient population and their family members were another workplace issue in various teaching hospitals. Because teaching hospitals are economically accessible to a low-income, non-local and less-educated patient population that is often unfamiliar with how a teaching hospital operates, conflicts can and do occur.

"The conflicts we face mostly occur due to encounter with the patient's companions because in this ward companions are not allowed in...yet they insist on accompanying the patient...which makes trouble for us...because we have to face the matron, supervisors and other staff in charge."

### Hospital management style

Participants believed that flaws in management styles at different levels contributed to conflict and its ineffective resolution. Authoritarian bearing, abuse of power, illogical actions and failure to support the staff were some of the weak points that participants recounted from their experiences. One participant provided this example:

"We told our problems to the supervisor and asked him to see to them. For example, I asked the supervisor to intervene but to my surprise not only didn't he help solve the problem, he added to it."

Participants contended that planning, clarifying objectives, supporting the staff, fairness, tending to staff rights and understanding the staff, along with other appropriate leadership measures, can have a significant role in controlling conflicts and preventing resignations and loss of motivation. Participants believed that some managers' behaviour influenced an increase in conflict occurrence. Some managers were seen to have mistreated staff, shown unreasonable behaviour, discriminated, suddenly changed style, failed to understand and support the staff, violated staff rights, aggravated conflict intensity, discouraged teamwork and ignored nurses' problems. Moreover, participants expressed some of their experiences for reduction of conflict through taking their concerns to upper management levels.

"We can't ignore the fact that heavy workload and shortage of skilled human resources affect our performance; despite our effort to get used to the situation, we are limited in coping. When you see that the supervisor stops backing us up and never steps into the ward to listen to us it makes us feel our rights have been violated."

"Now I see nobody is advocating for me as a nurse, I am alone on this ward up to this hour of the night and I need support... but who supports me?"

### The nature and conditions of job assignment

Another theme or category that emerged from data analysis was the nature and conditions of the job. Participants contended that this theme had double effects on the occurrence and control of conflict. Although nursing has always been regarded as a valuable and important profession, the current lack of professional regard for nurses has caused several internal and external conflicts. The importance of the work, responsibility, continuous contact with the patient, long working hours, night shifts, inadequate vacation time, high rate of staff turnover, heavy workload and excessive stress are all inherent to the nursing profession, affecting the threshold for rising conflicts.

"Most conflicts between my colleagues and me have been due to working shifts or hours clashing with our plans...arguing 'why does this colleague of mine have very light working hours but mine are so heavy?..."

"Well, if you are very exhausted, have been under pressure, have had a crowded shift, have been with patients all in bad conditions...sure you will develop conflict and an aggressive behaviour."

Therefore, it can be said that suboptimal working conditions can lead to exhaustion, mental pressure, tension and nervous breakdown, which in turn can result in leaves of absence and ultimately resignation, energy and motivation loss, and psychological problems for the nurses.

### Individual characteristics

The individual characteristics of participants involved specific situations at work where the potential source of conflict was more obvious and its resolution required management skills. These characteristics included an individual's personality, work commitment and moral characteristics. Any of these could play a role in creating or controlling conflict. Some of the participants recalled their experiences about the occurrence or control of conflict.

"Since I am a very easy-going person I rarely face conflict; I don't argue a lot."

"Conflict depends on the individual; there are some matters that may be important for me but not for others, or they may be important for others and not significant for me."

### Mutual understanding and interaction

Shared understanding and interaction was one of the most important categories. The majority of the participants regarded misunderstanding in interpersonal interactions as one important source of conflict. This inadequate mutual understanding occurs between nurses with other individuals and staff, such as patients, patient companions, managers and nursing and non-nursing colleagues at different position levels.

"I expect my manager to understand me...no matter if he does nothing for me...I just expect to hear a 'thank you', or 'yes, you're right on this, I understand you...it's a tough job, I know..."

"The patients' companions are not well informed ...their expectations don't fall into our area of responsibility...we can't meet their wants...it's difficult to make them understand that our services are directed at the patients not their companions."

Other factors that emerged from the collected data may increase or decrease this misunderstanding. Furthermore, the nature and conditions of the job, the teaching atmosphere and the structure of the hospital, management style and individual characteristics may have a double effect on this issue, thus improving or worsening the situation. Participants confirmed psychological stress arising from misunderstanding and emphasized the importance of mutual understanding between nurses and other staff.

Nevertheless, experiences of the participants indicated that they felt that patients and their families did not understand the nursing dilemma and work conditions. Also, participants emphasized the role of colleagues in the occurrence and intensity of conflict. These conflicts arose from sources such as doctors' influence on decision making, unwarranted interference of doctors and their inappropriate treatment of nurses. Other participants pointed out the role of colleagues other than doctors in the occurrence of conflict in clinical environments. Moreover, displacement of responsibilities onto nurses and other staff members who neglected their duties contributed to the occurrence of conflict.

"Patients have some expectation from us, but they don't understand that it isn't nursing duties. Today some drug must be purchased from outside of hospital. Many interns and residents visit patients, and nurses actually can't make any changes in these affairs."

Participants conceded that the existence of a cooperative environment could well prevent conflict, resolve the existing conflicts and prevent displacement of conflict onto hierarchical superiors. In all cases, participants agreed that mutual understanding and interaction can affect or be affected by other themes.

"We and our colleagues understand each other more, and we know that we have to work alongside each other peacefully, because if any tension is added, we may not be able to manage and control the working environment properly."

Expectations, viewpoints and cultures of the individuals were important from the participants' viewpoint. Expectations can definitely affect interpersonal interactions. Other highlighted issues were differences in cultures and belief that influenced conflict in the workplace.

"Conflict is meaningless in nursing because our primary purpose is caring for the patient to recover; so there should be no room for conflict."

### The consequences/outcomes resulting from conflict

An important category found in this study was the consequence/outcomes of the experience of conflict. Participants expressed several outcomes for conflict. Conflict can cause many psychological problems; agitation, loss of peace of mind, unhappiness, nervousness, sleep disorders and depression were identified by the participants. As well, conflict can lead to physical problems and occasionally the hospitalization of the affected individual.

"I remember once a patient's companion had such a blatant behaviour with me that I got hospitalized for the mental and nervous pressure inflicted on me...I got nervous breakdown."

In addition to these psychological and physical problems, the affected individual may lose motivation and become discontented, leading to indifferent and irresponsible behaviour at work and even a decision to resign.

"The main cause of our job dissatisfaction is these encounters and conflicts."

"When I experience a lot of stress I decide to change my job and choose another one, but when conflict is resolved I feel that I like my job and I love to work as a nurse."

Work-related outcomes are another aspect of this category. These consequences lie on a continuum with no outcomes on work at one end to resignation from work at the other. Some participants said that in their experience, despite existing conflicts, patient care was not affected and those internal conflicts did not affect meeting the patient's needs. Work-related outcomes of conflict are not limited to the individual; they also affect colleagues, managers and patients and their companions.

Some participants cited poor performance and neglect of patients as instances of unresolved conflicts. Other outcomes involve indifference or intolerance towards colleagues. Moreover, if not discharged progressively, the accumulated conflict can burst explosively and more destructively. Other experiences include disrupted performance, decrease in service quality, absenteeism, job dissatisfaction, forgetting care tasks, disrupted work routine, neglect of the patient, reluctant caring, conflict displacement onto the patient and a negative attitude towards the patient.

"Well...they are patients...and I know they need help...but sometimes you can't help it...those conflicts affect you...you get the feeling of discontent,...you don't work heartily...with reluctance...I give them the shots, serum, medicine...take their vital signs...all with reluctance and unwillingness."

"When I was in conflict with a patient or her/his companion, I couldn't focus on anything because I became nervous and I couldn't write a plain report, and my performance was affected."

Conflict can also affect the individual's family life. Participants' viewpoints ranged from lack of influence to adverse effects on family life. Displacement and inappropriate behaviour with family members and the disruption of the regular flow of life were some of the problems participants mentioned as having affected their family lives. They also suggested that nurses, during their education and training, be oriented about how to avoid transfer of work-related problems into the family.

"Surely it affects our lives...when you leave for home with a troubled mind you will make trouble for the family members...and this affects children and your whole life..."

## Discussion

The findings of this study reveal that issues such as the perception of and reaction to conflict, organizational structure, hospital management style, the nature and conditions of job assignment, individual characteristics, and mutual understanding and interaction are important factors contributing to the occurrence and control of conflict. Furthermore, the consequences/outcomes resulting from conflict were also discussed. Therefore, managers need to take these variables into account to increase efficiency.

In line with the findings of the current study, other research findings confirm the variability of the perception of and reaction to conflict as being affected by different variables. Jahoda and Wanless found that when facing conflict, employees would react with verbal or physical aggression such as yelling and hitting [19]. Researchers as well found relationship-destructive reactions such as criticism, faulting, humiliation, defensiveness and job resignation in conflict situations [20].

Organizational structure – such as the training nature of the hospitals, hospital equipment and facilities, hierarchy in organization, patients and patient companions – was another issue expressed in various ways by the participants. Other researchers have noted that competition for limited organizational resources can be a potential source of conflict [4]. When institutional priorities must be juggled against individual and departmental priorities in the face of limited time and other resources, conflict can result. Conflict increases with the number of levels in organizational hierarchy [4]. When employees work in very crowded settings, their interactions with colleagues and patients increase and potentially lead to stress, exhaustion, conflict and high turnover [2].

Research has also revealed the role of hospital management style adopted by managers in conflict control. Nelson and Cox found management approaches to be one of the conflict enhancers, contending that since autocratic managers try to prevent challenges and suppress conflict by force and coercion, they aggravate dysfunctional conflict [21].

The nature and conditions of job assignment, which was one of the major themes expressed by participants, has been investigated in various ways by different researchers. Cox and Kubsch concluded that task structures, task-based environments controlled by medical practitioners, group combination and size, and limited resources available to nursing managers can all function as conflict sources [22,23]. Overloading can lead to conflicts for most individuals [24]. One important strategy in reduction of conflict is a balanced nurse-patient ratio [21] and clear task descriptions [4]. Working conditions may bring about conflicts that induce nurses to resort to routine task performance, thus possibly negatively affecting health care, as is evident among Iranian nurses.

Regarding individual characteristics, we found that they are involved in the specific work situation as potential sources of conflict and its efficient resolution. Similarly, researchers contend that personal characteristics, attitudes and situational behaviors play significant roles in conflict issues [24].

Mutual understanding and interaction was found to be the most frequent and important category in the research, comprising different aspects such as mutual understanding between colleagues, managers, personnel, patients and patient companions. Other studies have shown that conflict can occur and be controlled through interactions and communication. Conflict arises because of misinformation or misunderstanding [21]. Inadequate communication between medical practitioners and nurses can lead to conflicts [25]. In his ultimate research model, Cox proposed that good personal interrelationships and a higher understanding of the spirit of others are negatively correlated with within-group conflict and can function as buffers [22].

Some participants believed that as nursing is a humanistic profession, conflict could not therefore affect nurses' performance. Cox did not find any direct relationship between conflict and performance and turnover [22], although some researchers [2,21,22,26] argued that a "good nurse should leave her/his personal life matters behind the hospital doors". However, by now it has been revealed that personal and life experiences can influence professional life and vice versa [27]. Further research has also revealed the outcomes of conflict on different individual aspects, the health of family life, poor performance and relationships, increase in patient care cost, imprecise and counterproductive care, and eventually an increase in turnover [2,21,26]. Generally it can be argued that not all the outcomes of conflict are negative; conflict can be constructive if it enhances decision-making quality [22].

Other finding in this study was that conflict can also affect the individual's family life. On the other hand, family life and multiple roles of the individual can also give rise to conflicts. Chandola et al. contend that both directions of conflict – work conflicts disrupting one's personal life and life conflicts disrupting work – affect health [26]. These conflicts can arise from the individual's inability to adopt multiple roles, which can lead to stress and illness. On other hand, conflict has arisen between nurses' perceived professional roles and the roles that the organization has imposed on nurses [28].

Organizational culture, task-oriented nursing experiences, unbalanced nurse-patient ratios and physician-centered organizations were found to be the main themes in other Iranian qualitative research [29-33]. Nikbakht found that Iranian nurses were confronted with many difficulties in two domains: (1) difficulties relating to work settings, such as personnel shortages, heavy workloads, unclear tasks, lack of registered and auxiliary nurses, equipment deficiencies and low salary; and (2) difficulties relating to a poor public image and a low social status of nurses [29]. Salsali also wrote that the role of nurses is unclear and largely unknown, even by the educated public [33]. It is clear that under these circumstances, the conditions that cause conflict are increased. Thus, nurses and nurse managers should be alert in order to prevent and control conflict effectively.

### Limitations

The main disadvantage of the qualitative approach is that the findings cannot be replicated for a larger population with the same degree of certainty that quantitative analyses provides. However, the results can be judged based on the criteria of transferability or applicability. This study provides a comprehensive understanding about factors that influence occurrence and control of organizational conflict. It is recommended that further research be carried out to explore conflict management in clinical settings.

## Conclusion

Iranian nurses experience conflict as a frequent incident in their work. According to our findings, how nurses perceive conflict influences how they behave or react concerning it. Conflict sources are embedded in nurses' and the nursing system's characteristics; at the same time, these characteristics can be considered as the strategies to resolve conflict. We found "mutual understanding and interaction" to be the main factor able to prevent and resolve conflict effectively. We therefore recommend that nurses and nurse managers encourage any virtues and activity that enhances such understanding and interaction. This approach will benefit the quality of patient care through a healthy work environment. Finally, as conflict can destroy individual nurses and the nursing system as a whole, it is advisable that we take action to control it effectively.

## Competing interests

The authors declare that they have no competing interests.

## Authors' contributions

NN planned the study, carried out the interviews, and carried out data analysis. RN and NN jointly developed an outline for the paper and wrote the initial draft, which they revised in accordance with comments from reviewers. Both authors have read and approved the final manuscript.
